# A spontaneously metastatic model of bladder cancer: imaging characterization

**DOI:** 10.1186/s12967-019-02177-y

**Published:** 2019-12-19

**Authors:** James L. Tatum, Joseph D. Kalen, Paula M. Jacobs, Lilia V. Ileva, Lisa A. Riffle, Melinda G. Hollingshead, James H. Doroshow

**Affiliations:** 1 Cancer Imaging Program, Division of Cancer Treatment and Diagnosis, National Cancer Institute, National Institute of Health, Rockville, MD USA; 2 Small Animal Imaging Program, Frederick National Laboratory for Cancer Research, Frederick, MD USA; 3 Biological Testing Branch, Developmental Therapeutics Program, Division of Cancer Treatment and Diagnosis, National Cancer Institute, National Institute of Health, Frederick, MD USA; 4 Division of Cancer Treatment and Diagnosis, and Center for Cancer Research, National Cancer Institute, National Institute of Health, Rockville, MD USA

**Keywords:** Patient derived xenograft models (PDXM), MRI, Metastasis, Non-clinical, Animal model, Bladder cancer

## Abstract

**Background:**

Spontaneously metastatic xenograft models of cancer are infrequent and the few that exist are resource intensive. In xenografts, caliper measurements can be used to determine primary tumor burden and response to therapy but in metastatic disease models determination of the presence of metastatic disease, metastatic burden, and response to therapy are difficult, often requiring serial necropsy. In this study we characterized the development of visceral metastases in a patient derived xenograft model (PDXM) using in vivo imaging.

**Results:**

We identified and characterized the previously unreported development of spontaneous liver and bone metastasis in a known patient derived xenograft, bladder xenograft BL0293F, developed by Jackson Laboratories and the University of California at Davis and available from the National Cancer Institute Patient-Derived Models Repository [1]. Among FDG-PET/CT, contrast-enhanced MRI and non-contrast MRI, non-contrast T2w MRI was the most effective and efficient imaging technique. On non-contrast T2 weighted MRI, hepatic metastases were observed in over 70% of animals at 52 days post tumor implantation without resection of the xenograft and in 100% of animals at day 52 following resection of the xenograft. In a group of animals receiving one cycle of effective chemotherapy, no animals demonstrated metastasis by imaging, confirming the utility of this model for therapy evaluation. There was good agreement between pathologic grade and extent of involvement observed on MRI T2w imaging.

**Conclusion:**

PDX BL0293F is a reliable visceral organ (liver) metastatic model with high penetrance in both non-aggravated and post excisional situations, providing a reliable window for therapy intervention prior to required excision of the xenograft. The imaging characteristics of this model are highly favorable for non-clinical research studies of metastatic disease when used in conjunction with non-contrast T2 weighted MRI.

## Background

The in vivo study of drugs and drug combinations in mouse models is invaluable in oncology drug development. The search for improved models that more closely mimic specific cancers in humans has led not only to an increasing number of models but also to diverse model strategies, from cell line derived xenografts to genetically engineered models. Recent efforts have focused on developing high quality patient derived xenograft models (PDXMs) that are low passage and well characterized in the hope of better recapitulating the characteristics of a patient’s tumor [2–4].

An even more daunting challenge has been the development of models of metastatic disease that could be employed for drug development. This most critical aspect of tumor biology is responsible for more than 90% of cancer deaths [5]. While all animal models are artificial simulations of human disease, most metastatic models are even less faithful to the clinical because they require either direct injection of tumor cells into the vascular system or aggravation by excision of the xenograft with subsequent local, lymphatic, and/or vascular contamination. Patient-derived orthotopic xenograft (PDOX) models that employ re-implantation of mouse derived xenografts into an orthotopic location may replicate some features of metastatic disease [6–8]. Metastatic models that do not require excision of the primary tumor, while uncommon, may be critical tools to understand this lethal aspect of cancer. A further challenge for metastatic models is that characterization of the metastatic disease can require sacrifice of numerous animals to determine the frequency, time course, metastatic tumor burden and, most importantly, response to therapy at metastatic sites. Longitudinal evaluation of response to therapy for metastases is not possible.

In this report we detail our imaging characterization of an established patient derived xenograft model (PDXM) of human bladder cancer [9] with previously un-reported metastatic potential and imaging characteristics favorable for non-invasive study of metastatic disease to the liver, both prior to and following excision of the primary tumor. The un-aggravated metastatic potential of this model was fortuitously discovered following an oncology drug study—survivors euthanized at the end of protocol treatment had liver and skeletal metastases on pathological examination. Non-invasive imaging permits serial monitoring of the same animals during treatment trials in this model system.

## Materials and methods

### Mouse model

Animal studies were performed according to the Frederick National Laboratory for Cancer Research (Frederick, MD) Institutional Animal Care and Use Committee guidelines (IACUC Protocol No. 14-004-M4).

Tumor fragments (8 mm^3^) were harvested from BL0293-F563 (The Jackson Laboratory, Bar Harbor, ME now designated TM00016; obtained from the NCI PDM repository, https://pdmr.cancer.gov/) tumor bearing mice and directly implanted into the right flank of female NOD-*scid* gamma (The Jackson Laboratory, Bar Harbor, ME) NSG mice.

### Imaging

**Fig. 1:**
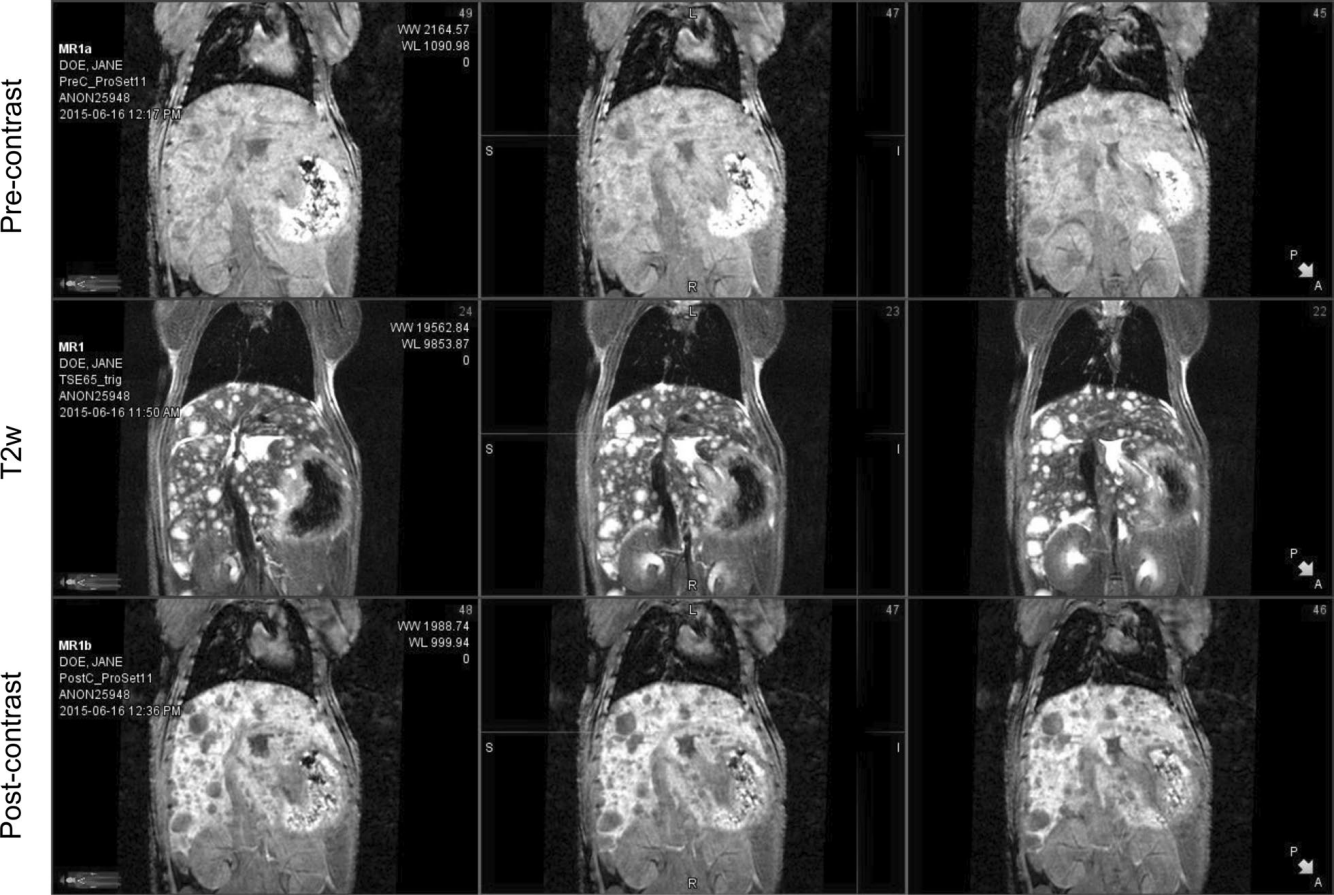
Comparison of MRI imaging of BL0293-F563 liver metastases. MRI images from a representative mouse with known BL0293-F563 metastatic disease to the liver comparing T1w non/pre-contrast images (top row), T2w non-contrast images (middle row) and T1w post contrast images (Eovist) bottom row. T2w imaging without contrast provides excellent MRI depiction of metastatic disease from this xenograft

**Fig. 2:**
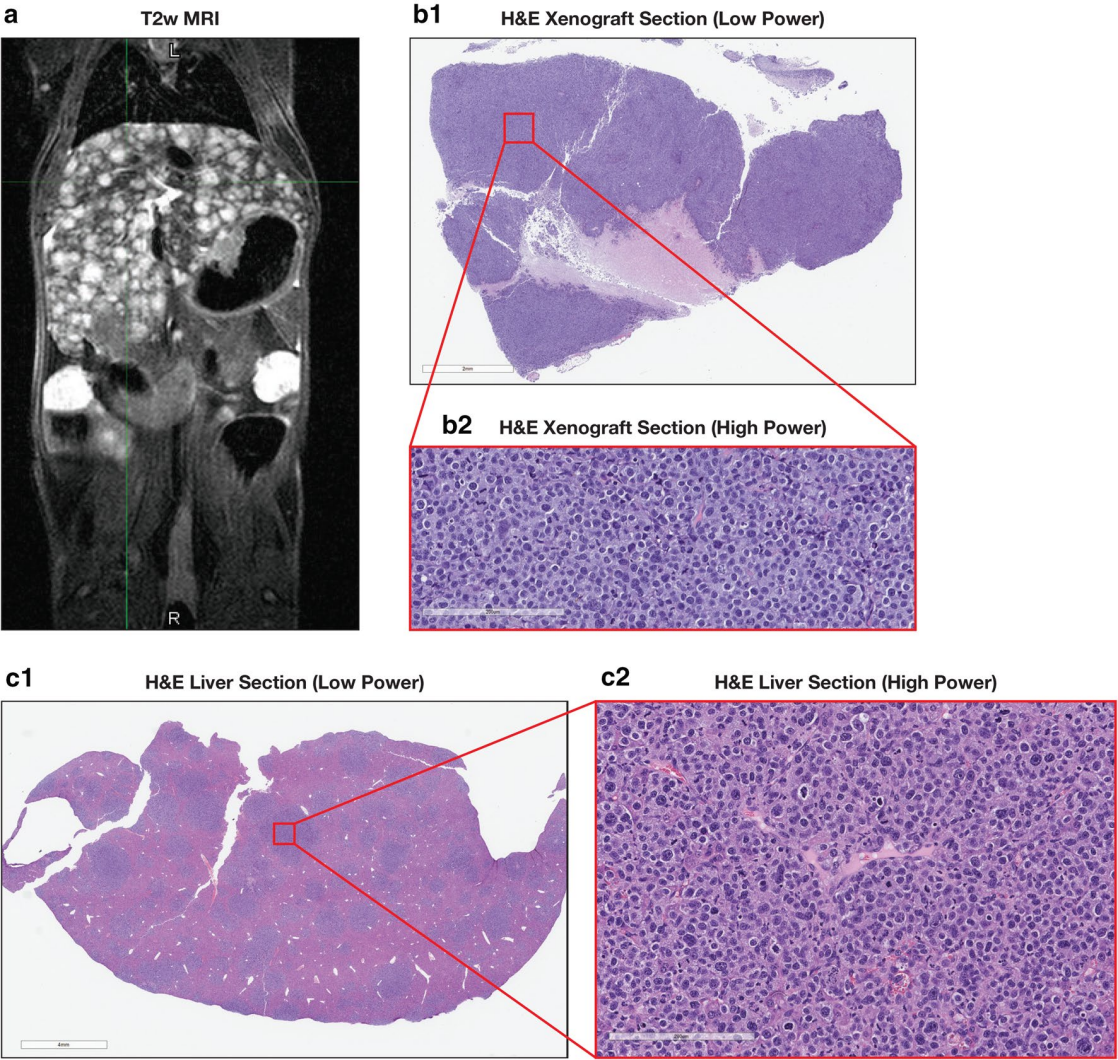
Pathologic confirmation of metastatic disease from xenograft. MRI of a representative mouse with imaging findings consistent with diffuse metastatic disease to liver (**a**). Section from the xenograft H&E low power (**b1**) with high power (**b2**). Metastases: low power H&E liver section (**c1**) and high power (**c2**)

Xenograft growth was measured approximately every 10 days with 3D-Ultrasound (US) (Vevo2100, VisualSonics, FUJIFILM Sonosite, Inc, Toronto, Ontario, CA) over a 2-month period. When the xenografts reached the enrollment criteria (100–200 mm^3^), advanced imaging was initiated consisting of 2-deoxy-2-(^18^F)fluoro-d-glucose ([^18^F]-FDG) positron emission tomography/computed tomography (PET/CT) (Inveon Multi-Modality PET/CT, Siemens Medical Solutions, Knoxville, TN) and T2 weighted MRI (Intera-Achieva 3T, Philips Healthcare, Best, The Netherlands) utilizing a 40 mm diameter mouse receiver coil (Philips Research, Hamburg, Germany). In a pilot MRI study, multiple acquisitions including non-contrast and contrast with gadoxetate disodium [(Eovist) (Bayer Healthcare Pharmaceuticals, Whippany, NJ), 0.05 mmol Gd/kg IV-tail vein injection] were performed in the same animals (Fig. 1). Non-contrast T2 weighted (T2w) MRI was determined to be optimal for imaging BL0293-F563 metastases. Standard H&E histology was used to confirm that hepatic metastases were of xenograft origin (Fig. 2).

### Animal preparation and physiology

The standard imaging and animal handling protocols for all modalities [10] required maintaining the rodent’s internal temperature and monitoring anesthesia administration. The animal body temperature (thermostat controlled heated table at 34–37 °C) was maintained from the time the animal entered the imaging room, through induction of anesthesia, imaging, and until recovery from anesthesia. Isoflurane anesthesia was administered via an induction chamber (3% pre-imaging) and nose cone (1.5–2% during imaging) with a carrier gas of either oxygen or filtered (0.2 µm) air at a flow of 1 l/min. Pulmonary function was monitored (MP100 and MP150, Biopac Systems, Inc, Goleta, CA) during scanning and the anesthesia (1.5–2% Isoflurane) was regulated to maintain a pulmonary rate between 50 and 90 breaths per minute for PET/CT and 40 bpm for MRI. Respiratory gating was implemented for MRI imaging to reduce motion artifact.

### PET/CT protocol

Mice were fasted (allowed water) for 8–12 h prior to [^18^F]-FDG injection (6.76 ± 1.25 MBq) via tail-vein injection [11]. PET-imaging commenced at approximately 1-h post injection. Mice were imaged in the prone position for a 12-min CT for PET attenuation correction, followed by a 20-min PET acquisition. CT acquisition parameters were: 80 kVp, 500 μA, 200 ms per step, 120 steps covering 220°. PET list-mode data were acquired using an energy window of 350–650 keV and a 3.432 ns coincidence timing window. CT images were reconstructed using a cone beam algorithm resulting in 192 × 192 matrix and PET utilized Ordered Subset Expectation Maximization (OSEM-3D) with 12 subsets and 4 iterations resulting in a 256 × 256 matrix.

[^18^F]-FDG PET/CT DICOM images were displayed and fused on a MIM workstation (v 6.6.5, MIM Software Inc, Cleveland, OH). Xenograft [^18^F]-FDG uptake was registered using a volume of interest (VOI) defined by CT, and the maximum standardized uptake value normalized by body weight (SUVbw max) was calculated using the same commercial software.

### MRI protocol

An initial MRI survey scan was acquired in three orthogonal planes (sagittal, coronal and transverse) which was used to determine the imaging planes and 3D volume [78 × 32 × 18 mm^3^; slice thickness (0.5 mm)]. A T2w turbo spin echo (T2w-TSE) sequence was applied in the coronal view with a repetition time (TR) 5333 ms, echo time (TE) 65 ms, with an in-plane pixel of 0.180 × 0.180 mm^2^. A Spectral Presaturation with Inversion Recovery (SPIR) sequence (Philips Healthcare, Best, The Netherlands) was used to suppress the fat component and assist in distinguishing fat from cystic mass and tumor tissue.

MRI DICOM images were displayed using MIM software. Images were re-oriented to conventional clinical radiological orientation and viewed on a MIM workstation in the 3 standard orthogonal views. Images were interpreted by a radiologist with experience in small animal imaging and graded using a standardized grading scale (Table 1) for semi-quantitative estimation of metastatic tumor burden. This MRI methodology generates high-resolution T2w images with improved contrast between the hyper-intense metastatic lesions and dark liver and lung tissues that allows for detection of metastases as small as ≤ 0.2 mm.

**Table 1:** T2w MRI metastases scoring system for BL0293

MRI score	Description
0	No evidence of metastases
1	Hepatic metastases (small) < 5
2	Hepatic metastases (large) < 5
3	Diffuse hepatic metastases
4	Hepatic metastases plus lung or bone
5	Hepatic metastases plus lung and bone
6	Diffuse metastases without bone
7	Diffuse metastases including bone

### Ultrasound protocol

Mice were prepared prior to imaging by shaving the area around the tumor using hair clippers and applying a depilatory cream (SurgiCream, American International Industries, Los Angeles, CA), followed by 1% acetic acid (pH of 2) to remove and neutralize the high pH of the depilatory. B-mode US images (Vevo2100, VisualSonics, Toronto, ON, CA) were acquired with a 40 MHz transducer (MS-550S, VisualSonics, Toronto, ON, CA) with an axial and lateral image spatial resolution of 40 and 90 µm, respectively. Heated gel (Aqua-Gel, Parker Laboratory, Inc., Fairfield, NJ) was applied to match the tissue acoustic characteristics between the animal and the transducer and the acoustic focus was placed at the center of the tumor.

3D-volumes were calculated using vendor supplied software (Vevo Lab v. 1.7.1, VisualSonics, Toronto, ON, CA) utilizing the parallel region-of-interest (ROI) method, as specified in the vendor manual.

### Validation of MRI for detection and quantitation of visceral (hepatic) metastases

While standard H&E histology was used to confirm that hepatic metastases were of xenograft origin (Fig. 2) this was not sufficient to validate that MRI imaging could be used as a quantitative imaging biomarker. The MRI scoring system (Table 1) for extent of disease for hepatic metastatic disease was validated by comparison with standard histological evaluation in 31 xenograft-bearing mice during the course of these studies. Following an MRI demonstrating liver lesions, animals were euthanized using carbon dioxide according to approved ACUC guidelines. At necropsy the liver was resected and fixed using formalin and tissue samples were embedded in paraffin. Slides of 5 µm microtome sections underwent routine Hematoxylin and Eosin staining. The extent of liver tumor involvement was estimated from the involved area as visualized on low power microscopy and rounded to nearest 10%.

## Therapy challenge study

**Fig. 3:**
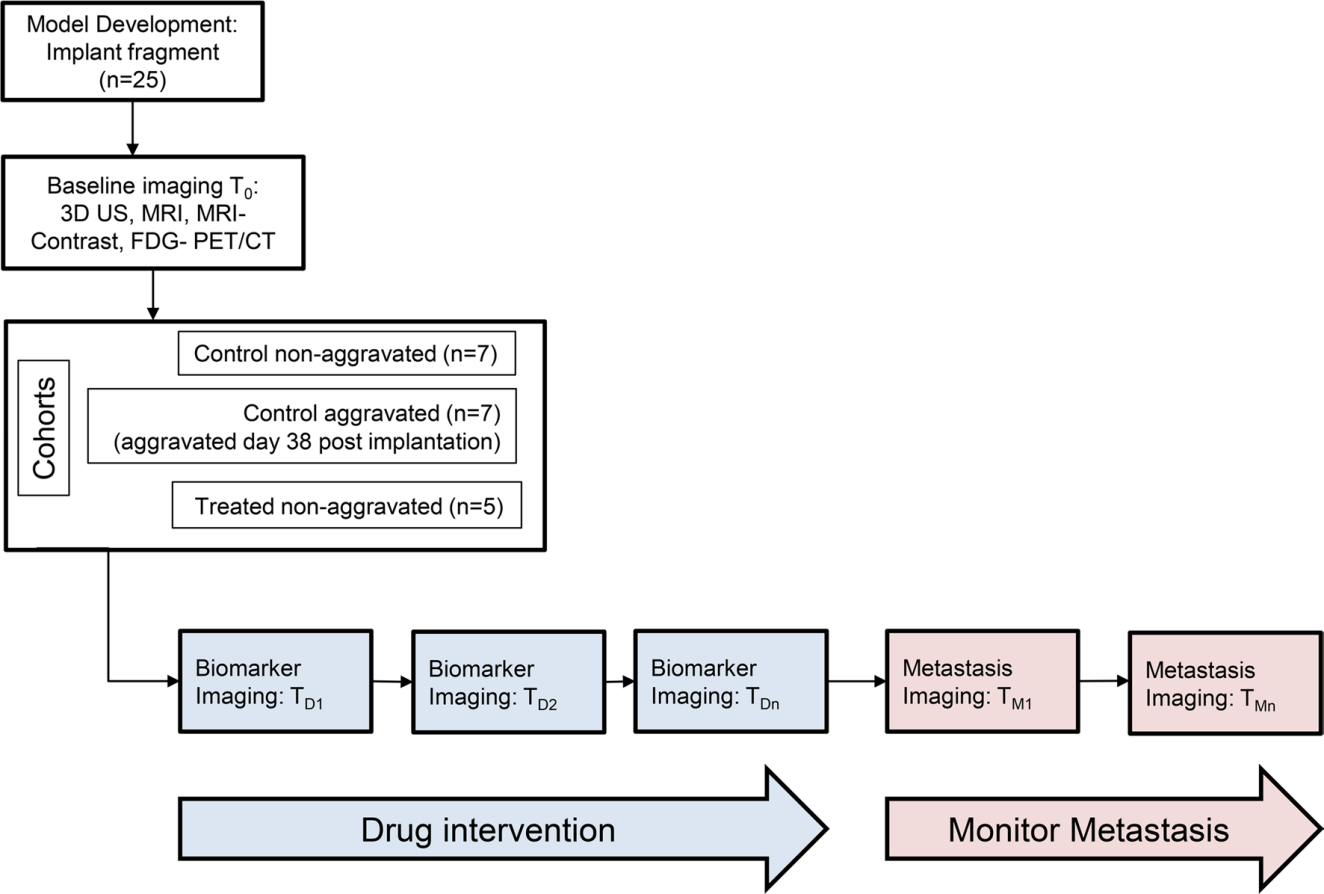
Imaging Characterization Schema. Schematic of BL0293-F563 Imaging Characterization Protocol

At 37 days post implantation, 19 of 25 mice had xenografts of sufficient pre-determined size for inclusion (100–200 mm^3^) and were randomized into three groups (Fig. 3). Seven (7) animals were designated control animals and underwent baseline FDG PET/CT and baseline and weekly T2w MRI until mice required euthanasia per ACUC guidelines (tumor dimension > 2 cm in any direction, or weight loss > 25%). These mice received no treatment and the xenograft was not excised (un-aggravated). Seven (7) animals were assigned to a surgical group and underwent the same imaging, but following baseline imaging the xenograft was excised. Another five (5) animals underwent the same imaging and comprised the drug challenge group. The primary tumors in these mice were not resected. These mice underwent a single cycle of drug treatment with a combination of agents known to be effective in this model [temozolomide, 50 mg/kg; PO, QDx5 plus veliparib, a poly (ADP-ribose) polymerase inhibitor, 7.75 mg/kg; PO; BIDx7] and were followed with MRI imaging to a protocol- determined endpoint of 122 days.

## Results

**Fig. 4:**
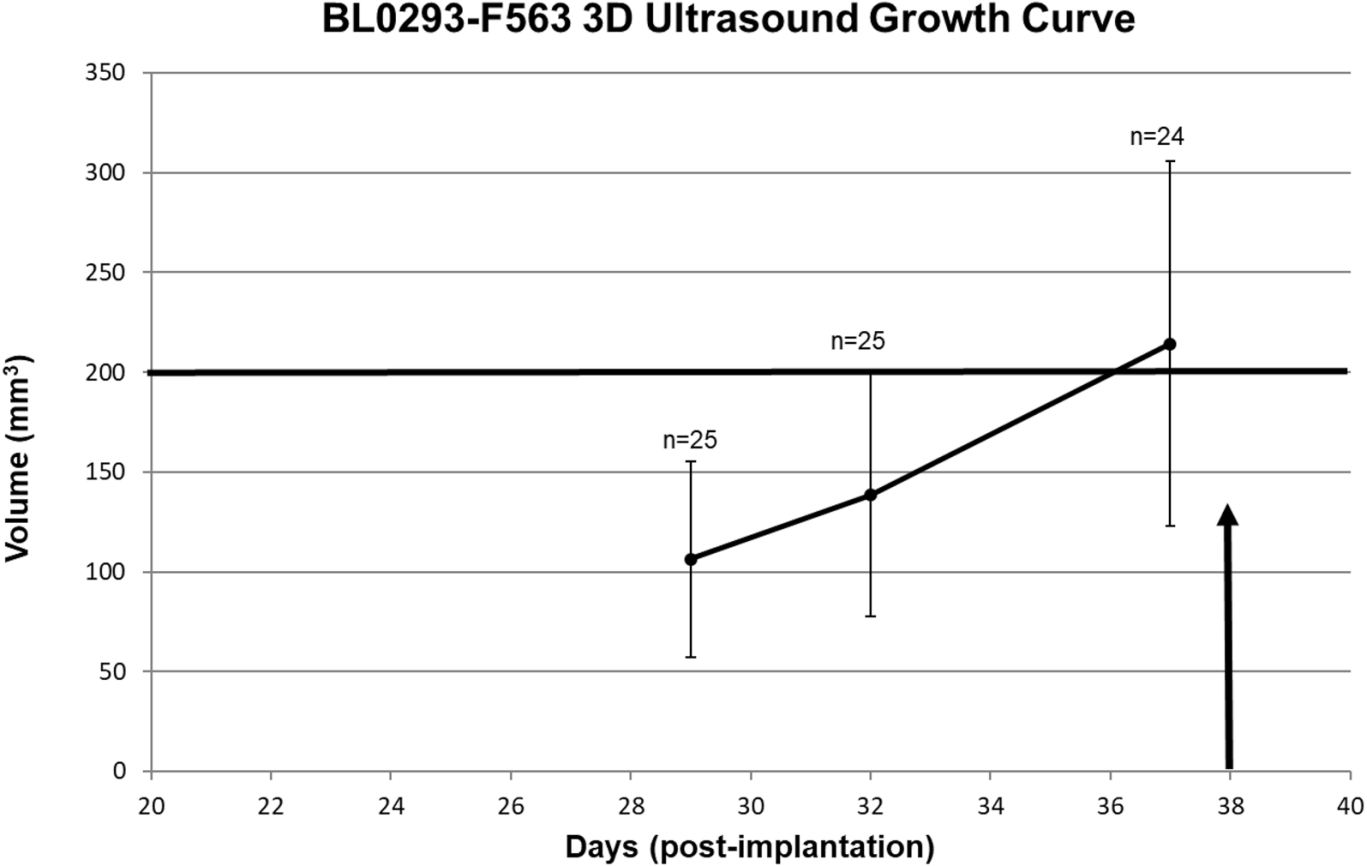
BL0293-F563 xenograft growth curve determined by 3D-Ultrasound. BL0293-F563 xenograft growth curve as measured by serial 3D-US. Mean time following implantation in right flank to desired volume of 200 mm^3^ was 37 days; however, the range was 50 to 300 mm^3^. The growth curve was consistent with previous caliper measurements

**Fig. 5:**
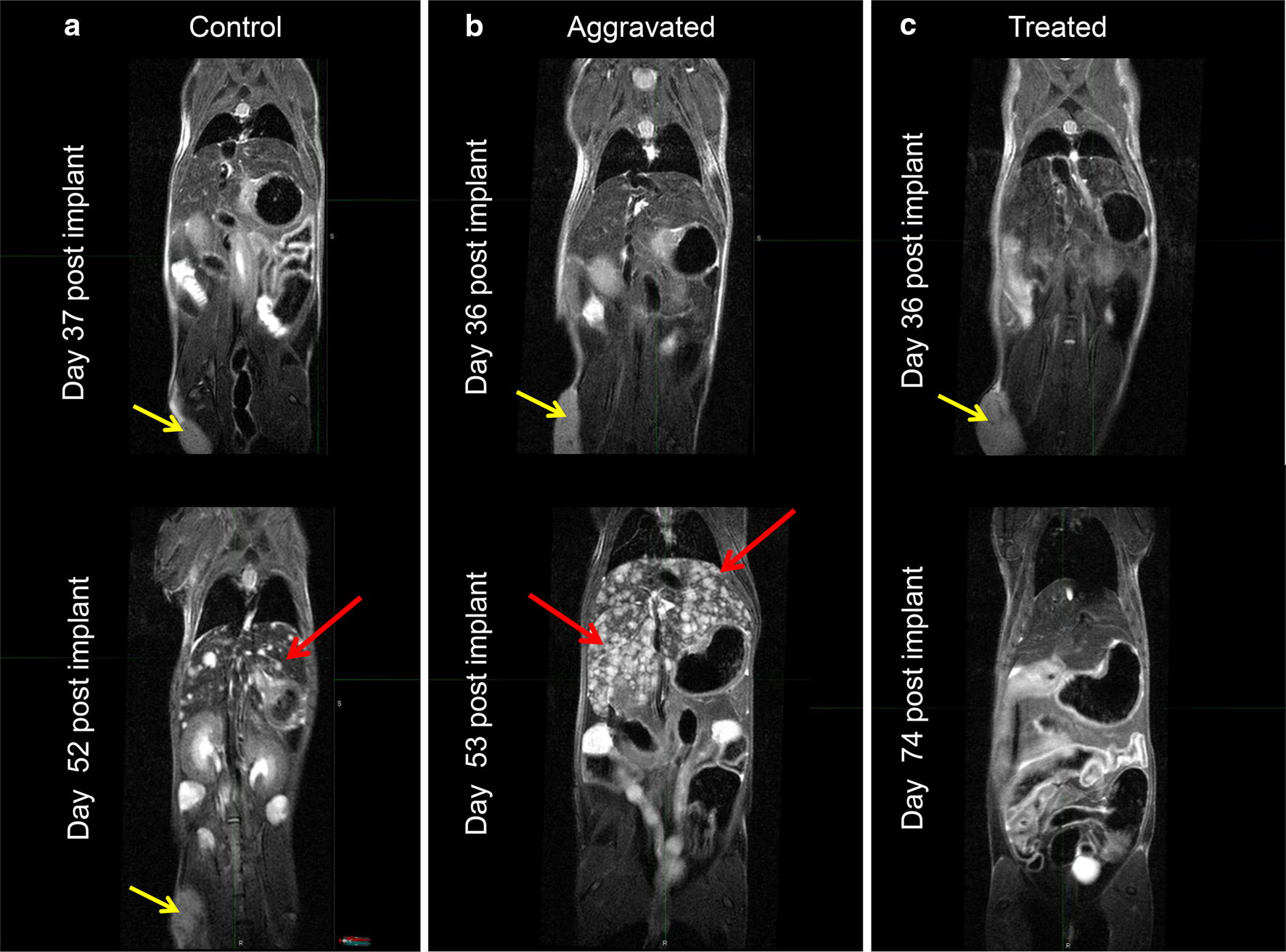
T2w MRI Coronal view including liver and xenograft. BL0293-F563 hepatic metastases as seen on MRI in a representative control animal without resection (**a**; left), aggravated (resected tumor) (**b**; middle), and an animal in the drug intervention group without tumor resection (**c**; right). Top row of coronal slices at day 36 post implant shows no evidence of metastatic disease on baseline MRI; however, 15 days later (52 days post implant) the same animal demonstrates numerous hepatic metastases in control and non-treated aggravated groups. The treated group demonstrated no evidence of metastases at day 74 and showed response of the primary tumor to the therapy (yellow arrows: BL0293 flank xenograft; red arrows: hepatic metastases). One animal in the drug treated group eventually developed hepatic metastases at 107 days, 2 weeks following a required excision of the xenograft post drug therapy due to ulceration

The 3D-Ultrasound (3D-US) studies demonstrated that the average xenograft reached approximately 200 mm^3^ at 37 days post implantation with a range of 101 mm^3^ to 445 mm^3^ and mean of 214.2 ± 91.4 mm^3^ (Fig. 4). The xenografts were [^18^F]-FDG positive at baseline with SUVbw (max): 4.9 ± 0.5. Metastatic disease was not present on the images at baseline MRI imaging (day 37–38 post implantation), prior to resection for the aggravated group. Control animals showed hepatic metastases on MRI in 5 of the 7 animals at 52 days post implant (Fig. 5a) with a tumor burden, estimated by the Scoring System (Table 1) of 2.7 ± 2.8. The primary xenograft size at the time of metastatic detection ranged from 245 to 589 mm^3^ with mean of 347 ± 153 mm^3^. The surgical group (aggravated) demonstrated evidence of metastatic disease in 7/7 animals at 14 days post resection (52 days post implant) (Fig. 5b) with an overall tumor burden at that time significantly greater than the control group with a score of 4.3 ± 2.1. The 5 animals in the drug intervention group showed no image abnormality (Fig. 5c), indicating no metastatic disease through 94 days and therefore had metastatic tumor burden score of 0. One (1) animal did demonstrate liver metastases at 107 days, 14 days after excision of xenograft because of tumor ulceration and a tumor score of 3 at 122 days.

**Fig. 6:**
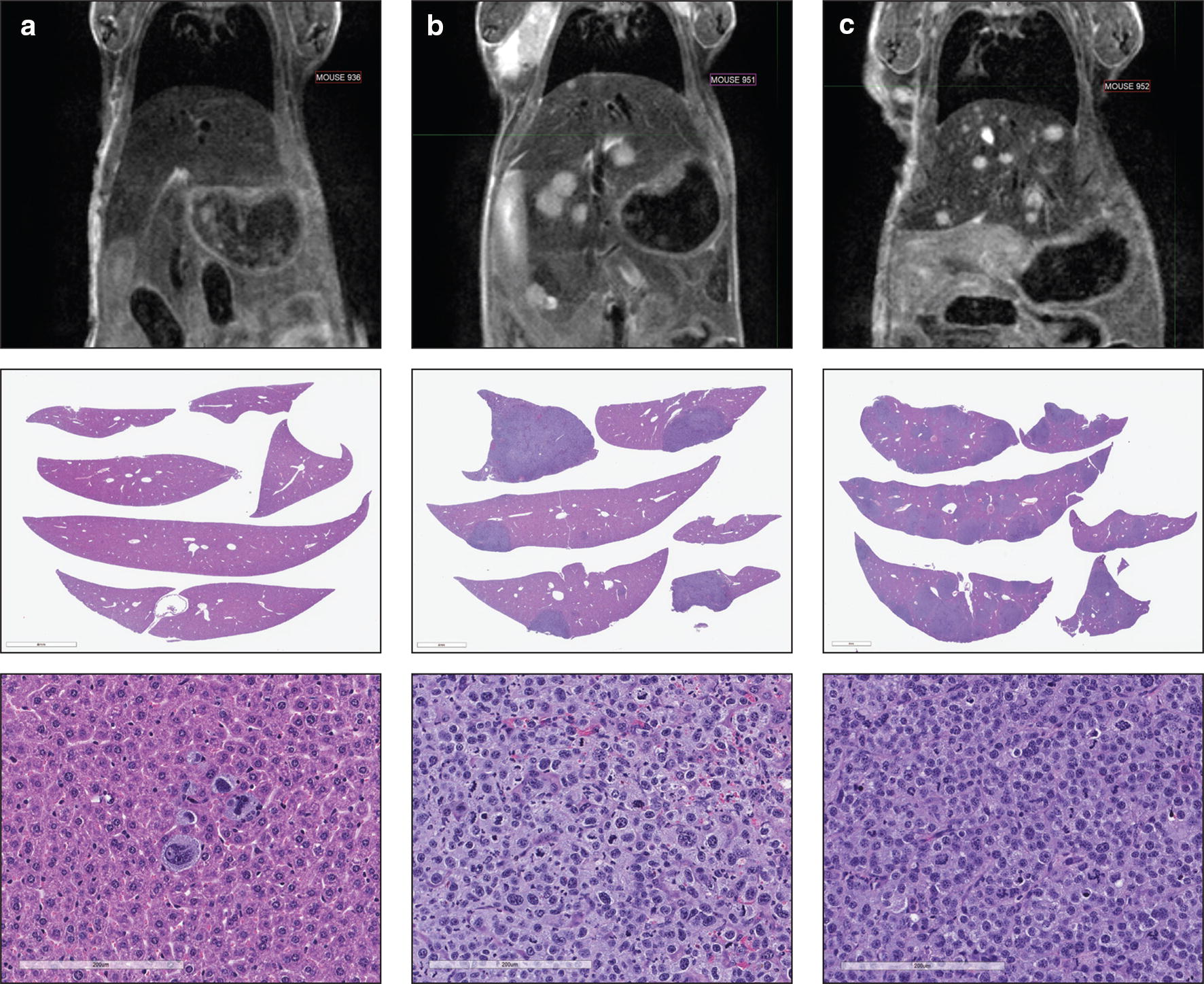
MRI image and histology correlations. Shown here are the MRI, low-power tissue image and high-power H&E images from a mouse with low (**a**), moderate (**b**) and severe (**c**) metastatic burden in the liver

In the pathology analysis to validate the imaging scoring system, animals displaying potential metastasis on MRI were confirmed by pathological analysis to have hepatic metastases of xenograft origin and imaging findings correlated with pathologic extent of disease. Figure 6 shows representative slides of histological grades of mild (0–20% hepatic involvement), moderate (30–50%) and severe (60–70%) with the corresponding MRI taken just prior to the histology evaluation. There was good agreement between pathologic grade and extent of involvement on MRI T2w imaging. MRI grade 2 hepatic metastases corresponded to an average 32 ± 13% hepatic involvement on pathologic correlation which corresponds to a pathology grade of Moderate; MRI grade 3 hepatic metastases correspond to an average 64 ± 11% hepatic involvement on pathologic correlation, corresponding to pathology grade of Severe.

## Discussion

Noteworthy progress has been made in understanding the complexity of the tumor cell relationship with the tumor micro-environment. However, our understanding of the complexity of the process and systems interaction between the macro-environment and tumor cells with metastatic potential is less well understood. Several studies have detailed the state of knowledge regarding the biology of this important process [12–14] and clearly support the need to target improved treatment, including early interventions to mitigate the metastatic process [15–17]. Developing effective treatment for primary malignancy has benefited from the availability of animal models, but, as previously noted [18], the paucity of high-quality animal models for metastatic disease is an unmet research need, despite the significant effort devoted to development of such models [19, 20]. Developing animal models of metastatic disease is challenging. The underlying systemic processes that drive some of the most popular aggravated (post-surgical) models remain poorly defined [21, 22]. While no metastatic model will perfectly mimic that of the human, metastatic models that disseminate without traumatic intervention would more closely resemble the human clinical situation.

The evaluation of metastatic disease in animal models is challenging. In xenografts, measurements of the primary implant with calipers or ultrasound can determine tumor burden and response to therapy. In metastatic disease models the presence, location, burden, and response to therapy of the metastatic deposits are not easily determined and require either serial necropsy or established in vivo imaging. In this study we characterized a PDX model with potential for development of visceral metastases. First, we determined that non-contrast T2w MRI was the most effective and efficient imaging technique among the readily available imaging modalities and protocols. We elucidated the characteristics of this model with serial MRI for penetrance, timing, tumor burden, and response to a known effective drug challenge.

BL0293 is one of several patient derived models of bladder cancer that were developed by the Jackson Laboratory (JAX) in conjunction with the University of California Davis (Davis, CA). The molecular characterizations of a number of these models including BL0293 have been published by the developers [9]. BL0293 was derived from a patient with myo-invasive bladder cancer who was naïve to previous treatment. It displays excellent stability both histologically and genetically over several passages. The un-aggravated metastatic potential of this model was fortuitously discovered following oncology drug testing. When survivors were euthanized at the end of a treatment protocol, routine pathology found liver and skeletal metastases. Because of the potential of this model for the investigation of metastatic disease, a series of imaging protocols was designed to evaluate the usefulness of serial imaging for repeated quantitative in vivo measurements that could be used in drug efficacy studies directed toward treating or preventing metastatic disease.

In pilot studies we demonstrated that hepatic metastases from BL0293-F563 could be easily visualized on non-contrast T2w MRI. This T2w MRI avoids the use of contrast agents with their associated morbidity and is suitable for serial imaging to follow each mouse over time. In these studies, we also determined that detectable metastatic disease was present relatively early, while the xenograft was still small, providing a practical window to begin drug testing without excisional aggravation.

The xenografts were [^18^F]-FDG positive, but no significant modulation of [^18^F]-FDG uptake was observed early in the drug challenge (day 3). An observed reduction at day 7 (end of the treatment cycle) was concurrent with significant reduction in tumor size on US and MRI, so FDG/PET is not needed to verify response. Additionally, the [^18^F]-FDG imaging protocol requires 8–12 h fasting prior to injection, causing significant mouse weight loss (> 25%). The high [^18^F]-FDG background in the liver further complicated visualization of hepatic metastatic disease with serial [^18^F]-FDG PET/CT.

MRI has the advantage of providing high quality images of the whole mouse as well as the xenograft, allowing estimation of the total tumor burden. MRI detected metastatic disease in a high percentage of control animals at 52 days following implantation. While we were primarily interested in un-aggravated metastatic disease, we did observe metastatic disease in 100% of animals (surgical group) at 14 days post xenograft excision. Overall this metastatic model provides excellent imaging characteristics for the study of interventions in vivo with repeated measures in the same animal, markedly reducing the number of animals required in the research protocol.

A subset of animals treated with an effective drug combination allowed evaluation of changes in imaging features to serve as response biomarkers. Compared to the control group, 0/5 mice in the drug challenge group showed evidence of metastatic disease at 94 days while 5/7 in the control group showed hepatic metastases at 52 days.

## Conclusions

While BL0293 has been characterized at the molecular level [9], in this report we also demonstrate that this model is an exceptional visceral organ (liver) metastatic model with high penetrance in both non-aggravated and post-excisional situations. In addition, the BL0293 xenograft growth characteristics and reliable timing of metastatic disease development provide a window for therapy intervention prior to required excision of the xenograft. Combining these characteristics with our demonstration that non-contrast T2w MRI is a robust non-invasive method for the detection and quantification of hepatic metastatic disease in this model provides a reliable non-invasive method for detection and quantitation of hepatic metastatic disease. Because of the low morbidity and lack of radiation, serial T2w non-contrast imaging can be used as a response marker for therapeutic efficacy. However, T2w imaging characteristics of tumors vary, so each new model will require similar demonstration of imaging characteristics to be used in drug development.

## Data Availability

The datasets generated and analyzed in the current study are available in The Cancer Imaging Archive (TCIA): https://wiki.cancerimagingarchive.net/pages/viewpage.action?pageId=52757379. There are no restrictions on the use of this data.

## Abbreviations

DICOM: Digital Imaging and Communications in Medicine
[^18^F]-FDG: 2-deoxy-2-(^18^F)fluoro-d-glucose
MRI: Magnetic Resonance Imaging
NSG: NOD-*scid* gamma
PDX: Patient Derived Xenograft
PDXM: patient derived xenograft model
PDOX: patient-derived orthotopic xenograft
PET/CT: positron emission tomography X-ray computed tomography
SUVbw (max): maximum standard uptake value (body weight normalized)
3D-US: 3-dimesional Ultrasound
VOI: volume of interest
